# The Development and Preparation of Novel Gel Emulsion Systems Based on a Cholesterol Star-Shaped Derivative

**DOI:** 10.3390/molecules30040787

**Published:** 2025-02-08

**Authors:** Shuaihua Liu, Tian Yao, Donghui Xia, Quan Liu, Guanghui Tian, Yang Liu

**Affiliations:** 1 School of Chemistry and Environment Science, Shaanxi University of Technology, Hanzhong 723001, China; liushuaihua@snut.edu.cn (S.L.); yaotian@snut.edu.cn (T.Y.); dhxia@snut.edu.cn (D.X.); liuq@snut.edu.cn (Q.L.); tiangh@snut.edu.cn (G.T.); 2 Shaanxi Key Laboratory of Catalysis, Shaanxi University of Technology, Hanzhong 723001, China

**Keywords:** low-molecular-mass gelators, stabilizer, polymerizable gel emulsions

## Abstract

Low-molecular-mass gelators (LMMGs) as stabilizers for gel emulsions offer numerous advantages, such as low usage, functionalizability, and insensitivity to phase ratio. Using LMMGs as stabilizers is one of the effective strategies for preparing gel emulsions. Currently, developing LMMGs and stable gel emulsion systems in a rapid and convenient manner remains a challenge. To cope with the challenge, this study aims to develop a simple and efficient gel emulsion preparation method based on LMMGs. In this study, a cholesterol-based star-shaped derivative (CSD) was designed and synthesized as an LMMG. Based on gelation experiments, a high internal phase W/O gel emulsion system (H_2_O/CSD-poly(oligo)-dimethylsiloxane/dichloromethane) was successfully developed and stabilized synergistically by the stabilizer (CSD) and the crosslinker (poly(oligo)-dimethylsiloxane with two olefinic bonds at its ends, D-PDMS). The results demonstrate that the synergistic interaction between CSD and D-PDMS is critical for the formation of the gel emulsion. Building on the original gel emulsion system, two novel in situ polymerizable gel emulsion systems (H_2_O/CSD-D-PDMS/dichloromethane-*tert*-butyl methacrylate and H_2_O/CSD-D-PDMS/dichloromethane-*N*-*tert*-butyl methacrylamide) were successfully developed by introducing suitable amphiphilic (hydrophilic/lipophilic) polymerizable monomers. This study found that changes in the amphiphilicity of the introduced monomers significantly affected the stability and microscopic morphology of the gel emulsion system. The findings indicate that constructing a hydrophilic/lipophilic balanced system via the synergistic action of stabilizers and crosslinkers in a solvent system, followed by the introduction of polymerizable monomers, is a simple and efficient method for rapidly developing novel polymerizable gel emulsions. These new polymerizable gel emulsions lay the foundation for the subsequent preparation of porous organic polymers (POPs).

## 1. Introduction

An emulsion is a multiphase dispersed system formed by mixing two immiscible liquids (typically oil and water), where one liquid is dispersed as small droplets within the other. The liquid present in droplet form is referred to as the dispersed phase (internal phase), while the liquid surrounding these droplets and forming the main body of the emulsion is known as the continuous phase (external phase) [1,2]. In addition, stable emulsions typically require the addition of a third component—an emulsifier—which can significantly reduce the interfacial tension between the oil and water phases, increase the viscosity of the system, and hinder the aggregation of the dispersed phase, thereby making the system relatively stable. The basic types of emulsions generally include oil-in-water (O/W) and water-in-oil (W/O), which have been widely applied in various fields such as cosmetics, pharmaceuticals, food, materials, and aerospace [3,4].

Gel emulsions are a special type of emulsion that combines the characteristics of both emulsions and gels. They are composed of a stabilizer, a continuous phase, and a dispersed phase. Regular emulsions typically have low viscosity and can flow under the influence of gravity. In contrast, gel emulsions lose their fluidity due to the closely packed droplets of the dispersed phase and the three-dimensional network structure formed by the self-assembly or aggregation of stabilizers under specific conditions, which maintains the continuous phase. As a result, gel emulsions exhibit higher viscosity and excellent stability [5,6,7]. Gel emulsions appear similar to solids or semi-solids and possess certain elasticity and mechanical strength. Even when subjected to some degree of gravity, shear force, or temperature changes, they can still maintain relative stability. In most cases, the volume fraction of the dispersed phase in gel emulsions can reach over 90%, which is why they are also referred to as high internal phase emulsions or viscous emulsions [8,9]. Currently, gel emulsions can be classified based on their dispersion type into various categories, including water-in-oil (W/O) gel emulsions [10], oil-in-water (O/W) gel emulsions [11], and multiple gel emulsions (W/O/W or O/W/O) [12], among others. Gel emulsions can also be classified into three types based on the stabilizer used: those stabilized by solid nanoparticles (micro/nano-particles), surfactants, and low-molecular-mass gelators (LMMGs) [13,14,15]. In the three types of gel emulsions mentioned later, the first two types can only achieve stable gel emulsions when the volume fraction of the dispersed phase is greater than 74% [16]. Additionally, gel emulsions stabilized by nanoparticles are prone to phase inversion [17]. However, when LMMGs are used as stabilizers for gel emulsions, they can self-assemble into fiber network structures through their weak interactions (such as hydrogen bonding, π–π stacking, van der Waals forces, electrostatic interactions, etc.) and encapsulate the dispersed phase droplets. This not only enhances the viscosity of the system but also prevents phase separation caused by droplet aggregation, significantly improving the stability of the gel emulsion [18]. In addition, LMMGs can efficiently stabilize gel emulsions at low dosages, reducing the system’s toxicity. Importantly, the volume fraction of the dispersed phase can be adjusted over a wide range, without being limited to 74%, and phase inversion is less likely to occur. These advantages are beneficial for the development of their applications in various fields [19,20]. Due to the aforementioned advantages, LMMGs are often popular and preferred stabilizers for the development of gel emulsions. However, the rapid and convenient development of LMMGs and stable gel emulsion systems still presents significant challenges.

Studies have shown that cholesterol derivatives, such as LMMGs, exhibit excellent performance in the development of gel emulsions [21,22]. Cholesterol, with its multiple chiral centers, rigid structure, and strong intermolecular interactions, is often used as an important structural unit in LMMGs. Cholesterol derivatives, similar to cholesterol, possess advantages such as easy self-assembly, high stability, good biocompatibility, and tunable structures, which have long been valued by chemists [23]. Additionally, literature reports indicate that star-shaped conjugated small molecules also demonstrate outstanding performance in the development and application of porous organic polymers (POPs) [24,25]. Star-shaped conjugated small molecules refer to a class of molecules with a structurally symmetrical central core, from which three or more conjugated arms radiate outward, resembling a “star” or “radial” shape. The central core can be a benzene ring, triazine, benzothiazole, etc. Due to their high degree of branching, strong rigidity, and ease of synthesis, star-shaped conjugated small molecules can impart unique properties and preparation advantages to materials. Gel emulsions stabilized by these small molecules are widely used in the development of materials for applications such as luminescence and adsorption [26,27,28].

**Figure 1:**
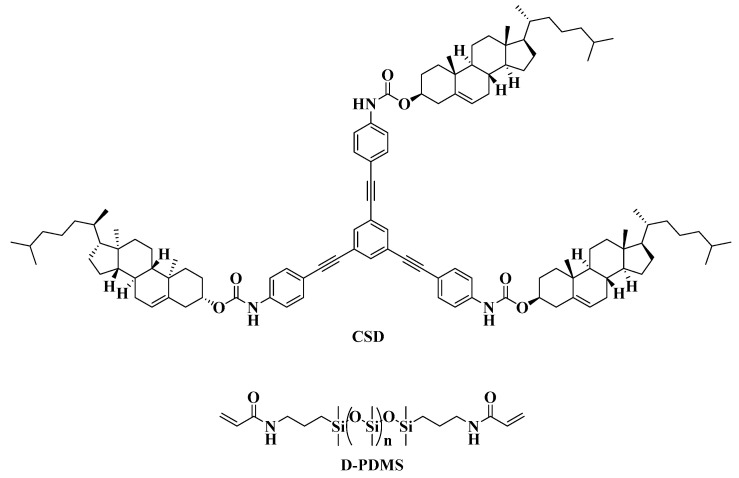
Structure of CSD and D-PDMS.

Based on the structural characteristics and properties of cholesterol derivatives and star-shaped conjugated small molecules, a cholesterol star-shaped derivative (CSD, Figure 1) was designed and synthesized in this study as an LMMG. To obtain a polymerizable gel emulsion system, D-PDMS (Figure 1), which has excellent physicochemical properties and two vinyl groups at both ends, was selected as the crosslinker [22]. Initially, a high internal phase gel emulsion system stabilized synergistically by CSD and D-PDMS was developed. Subsequently, to ensure that the POPs obtained through emulsion polymerization exhibit a rich three-dimensional network structure, a series of polymerizable monomers with a double bond were introduced into the original gel emulsion system as crosslinking points. On the basis of maintaining the stability of the gel emulsion system, two novel polymerizable gel emulsion systems were successfully developed. These novel polymerizable gel emulsions can be used for the next step in the preparation of corresponding POPs via a templating method.

## 2. Results and Discussion

### 2.1. Gelation Behaviors

The gelation behavior of CSD in 33 solvents was tested at a concentration of 3.5% (*w*/*v*), and the results are shown in Table 1. CSD was unable to gel any of the tested solvents at room temperature. However, under ultrasonic conditions, it could gel most organic solvents, which are immiscible with water. It is well known that LMMGs can gel certain organic solvents that are immiscible with water. Therefore, systems composed of water, LMMGs, and organic solvents have the potential to form gel emulsions [29].

### 2.2. Exploration of Gel Emulsion Systems

**Figure 2:**
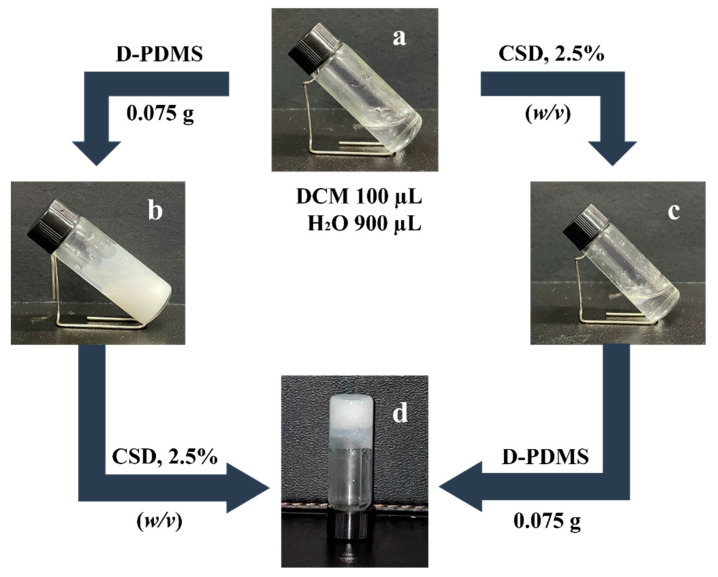
Gelation behavior of CSD in different components: (**a**) CSD 0% (*w*/*v*), 100 µL DCM, 900 µL water; (**b**) CSD 0% (*w*/*v*), 0.075 g D-PDMS; (**c**) CSD 2.5% (*w*/*v*); (**d**) CSD 2.5% (*w*/*v*) and 0.075 g D-PDMS (total volume 1 mL; CSD concentration is the mass volume fraction relative to the organic phase).

Based on the above gelation experimental results, the gel emulsion systems were explored. The experiments revealed that only the DCM/H_2_O (*v*/*v*, 1/9) solvent system could form a stable gel emulsion at room temperature (20 °C) when CSD was used as a stabilizer, while other ratios (9/1, 8/2, 7/3, 6/4, 5/5, 4/6, 3/7, 2/8) failed to form gel emulsions. This phenomenon suggests that the formation of the gel emulsion depends on the close packing of dispersed phase droplets [16]. To achieve a high internal phase gel emulsion system with minimal organic solvents and small-molecule gelators, the DCM/H_2_O (*v*/*v*, 1/9) solvent system was selected for the exploration and preparation of polymerizable gel emulsion systems. To enable the subsequent fabrication and development of POPs through the template method, D-PDMS, with its excellent physicochemical properties, was chosen as the cross-linker for the gel emulsion system [21]. As shown in Figure 2, when only DCM and H_2_O were present without the addition of a stabilizer, the solvent system remained clear (Figure 2a). Upon introducing 2.5% (*w*/*v*, relative to the organic phase) of CSD and shaking the mixture in a homogenizer, the solution became slightly turbid but did not form an emulsion (Figure 2c). This indicates that CSD is not an effective emulsifier for this solvent system. To achieve a stable gel emulsion system, a substance capable of emulsifying the solvent system must be added. The experiment revealed that the synthesized crosslinker D-PDMS could emulsify the solvent system (Figure 2b). Therefore, by adding an appropriate amount of D-PDMS to the original system and shaking it uniformly, a stable gel emulsion could be obtained (Figure 2d). The resulting gel emulsion had an internal phase volume fraction of 90%, classifying it as a high internal phase gel emulsion, and it maintained its shape at room temperature (20 °C) for at least one month. Furthermore, the gel emulsion could also be formed by adding D-PDMS first, followed by CSD. The experiment demonstrated that neither CSD nor D-PDMS alone could form a gel emulsion; only the simultaneous use of both components resulted in a stable gel emulsion. The reason for this experimental phenomenon is believed to be as follows: (1) Based on the molecular structure characteristics and experimental phenomena, it can be inferred that the lipophilicity of CSD dominates, which is primarily expressed in its oil solubility. (2) D-PDMS, due to its ether bond structure, can form hydrogen bonds with water, making it amphiphilic. This amphiphilicity may provide phase transfer functionality [30,31], allowing hydrophobic CSD to be transferred to the oil–water interface, promoting the formation of a hydrophilic/lipophilic balance between CSD and D-PDMS at the oil–water interface in the solvent system, ultimately resulting in the formation of a gel emulsion. In other words, only the synergy between CSD and D-PDMS can provide a stable functionality similar to LMMG, enabling the system to form a gel emulsion.

### 2.3. Microscopic Morphology of the Gel Emulsion

**Figure 3:**
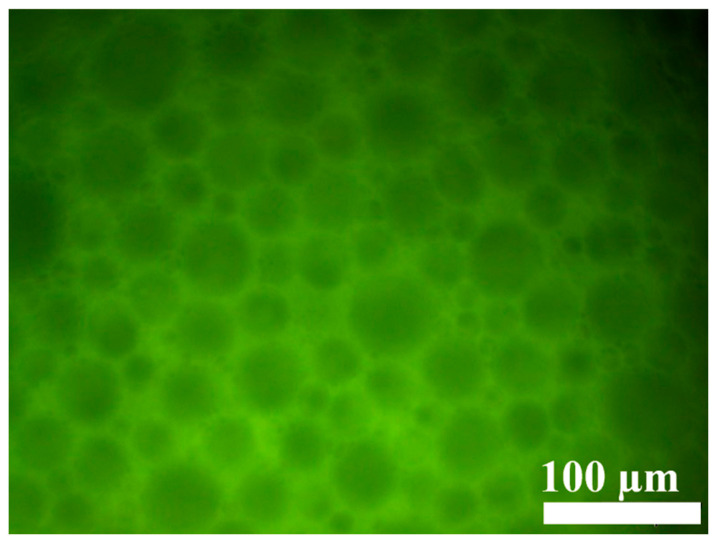
Inverted fluorescence microscopy image of the gel emulsion, with the following components: 2.5% CSD (*w*/*v*, mass volume fraction relative to the organic phase), 0.0175 g D-PDMS, 100 µL DCM, 900 µL water.

The microscopic morphology of the gel emulsion was observed using an inverted fluorescence microscope. As shown in Figure 3, the microstructure of the gel emulsion is consistent with that reported in the literature, exhibiting a typical binary liquid bubble film structure [32,33,34]. From the fluorescence microscope images, it can be seen that the fluorescence in the gel emulsion system originates from the layers between the droplets. This indicates that the mixture of DCM and D-PDMS forms the continuous phase, while water acts as the dispersed phase, resulting in a typical W/O-type gel emulsion.

### 2.4. The Effects of Different Polymerizable Monomers on Gel Emulsions

**Figure 4:**
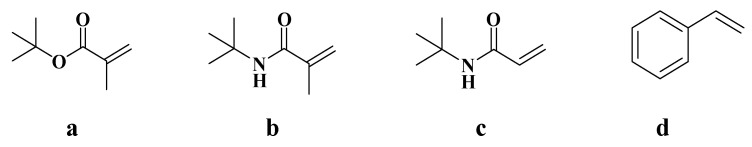
Four polymerizable monomers: (**a**) *tert*-butyl methacrylate (*t*-BMA), (**b**) *N*-*tert*-butyl methacrylamide (*N*-*t*-BMA), (**c**) *N*-*tert*-butyl acrylamide (*N*-*t*-BAA), (**d**) styrene.

After obtaining a stable high internal phase gel emulsion system, this study explored new gel emulsion systems capable of in situ polymerization. Four commonly used polymerizable monomers were introduced into the original system as crosslinking points, *tert*-butyl methacrylate (*t*-BMA), *N*-*tert*-butyl methacrylamide (*N*-*t*-BMA), *N*-*tert*-butyl acrylamide (*N*-*t*-BAA), and styrene (Figure 4), with the aim of developing a novel gel emulsion system that can be used to prepare corresponding POPs via in situ polymerization.

**Figure 5:**
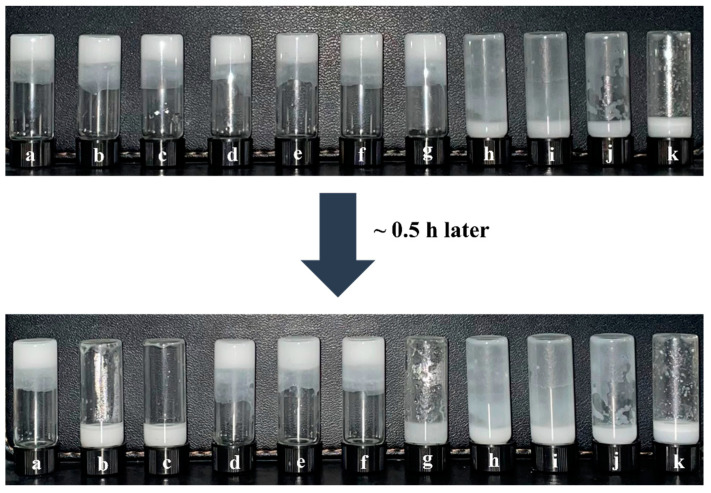
Photos of gel emulsions formed with different DCM–*t*-BMA (*v*/*v*) ratios: (**a**) 10:0, (**b**) 9:1, (**c**) 8:2, (**d**) 7:3, (**e**) 6:4, (**f**) 5:5, (**g**) 4:6, (**h**) 3:7, (**i**) 2:8, (**j**) 1:9, (**k**) 0:10 (stabilizer content is 2.5% (*w*/*v*) of the organic phase, D-PDMS is 0.075 g, and the aqueous phase makes up 90% of the total volume).

**Figure 6:**
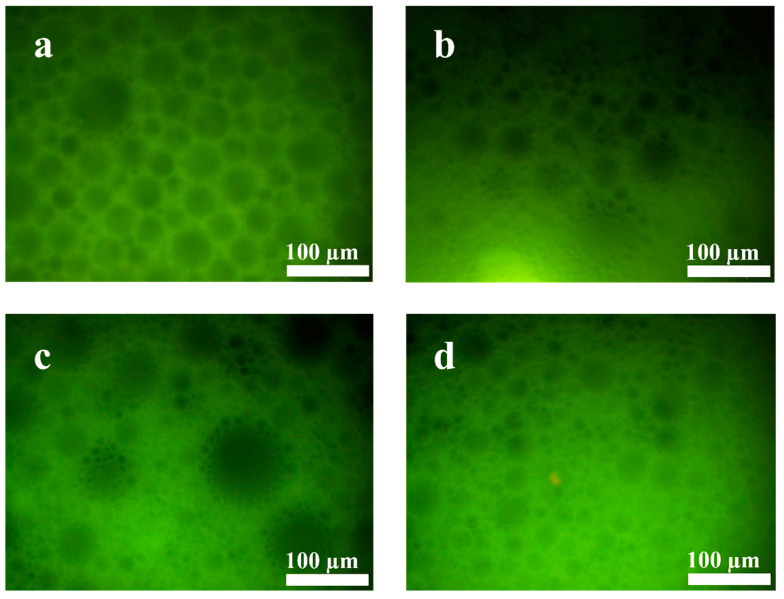
Inverted fluorescence microscope images of gel emulsions formed with different DCM–*t*-BMA (*v*/*v*) ratios: (**a**) 10:0, (**b**) 7:3, (**c**) 6:4, (**d**) 5:5.

Figure 5 shows that the new gel emulsion system formed by adding *t*-BMA to the original system still exhibits good stability. This is likely because the hydrophilic/lipophilic amphiphilicity of *t*-BMA is relatively suitable, causing no significant disruption to the hydrophilic/lipophilic balance of the original gel emulsion system. Nevertheless, as *t*-BMA is added in different proportions, the stability of the gel emulsion system shows a parabolic trend. At room temperature (20 °C), gel emulsion systems with DCM–*t*-BMA ratios of 9:1, 8:2, and 4:6 remain stable for only a relatively short period (~0.5 h), whereas systems with DCM–*t*-BMA ratios of 7:3, 6:4, and 5:5 remain stable for up to one month. Although the gel emulsion systems with DCM–*t*-BMA ratios of 7:3, 6:4, and 5:5 achieve relatively stable structures, observations from the inverted fluorescence microscope reveal that their microstructures are significantly inferior to those of the original gel emulsion system (Figure 6). It was observed that some degree of droplet coalescence occurred in the new gel emulsion system, indicating the onset of Ostwald ripening (Figure 6). Nevertheless, *t*-BMA possesses amphiphilic properties due to its ester group forming hydrogen bonds with water molecules. This amphiphilicity plays a crucial role in establishing a new hydrophilic/lipophilic balance in the gel emulsion system. Therefore, by adding an appropriate amount of *t*-BMA to the original gel emulsion system, a relatively stable new system can still be formed.

**Figure 7:**
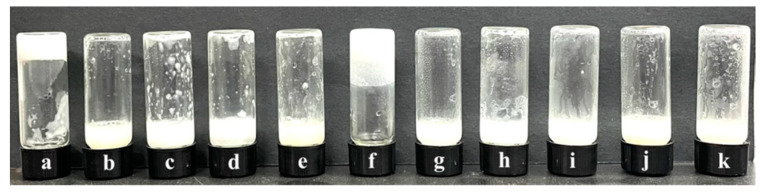
Photos of emulsions or gel emulsions formed at *N*-*t*-BMA concentrations ranging from 0% to 100% of the saturation concentration (relative to the organic phase): (**a**) 0%, (**b**) 10%, (**c**) 20%, (**d**) 30%, (**e**) 40%, (**f**) 50%, (**g**) 60%, (**h**) 70%, (**i**) 80%, (**j**) 90%, (**k**) 100% (stabilizer content is 2.5% (*w*/*v*) of the organic phase, D-PDMS is 0.075 g, and the aqueous phase makes up 90% of the total volume).

**Figure 8:**
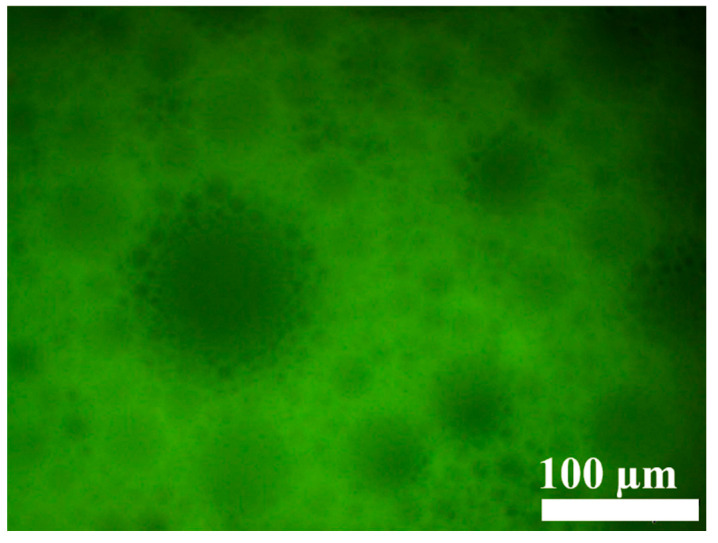
Inverted fluorescence microscope image of the gel emulsion formed at 50% of the saturation concentration (relative to the organic phase) of *N*-*t*-BMA.

Subsequent inversion experiments (Figure 7) revealed that the gelation performance of the new system formed after adding *N*-*t*-BMA was significantly worse than that of the system with *t*-BMA (Figure 5). A gel emulsion could only be formed when the concentration of *N*-*t*-BMA reached 50% of its saturation concentration (relative to the organic phase), and the system maintained its shape at room temperature (20 °C) for only about one week. It is noteworthy that the structural difference between these two polymerizable monomers is minimal (Figure 4a,b), yet this slight structural variation may result in the hydrophilic/lipophilic balance of *N*-*t*-BMA being less ideal than that of *t*-BMA, which caused some degree of disruption to the stability of the original gel emulsion system. Observation of the microscopic morphology of the new gel emulsion system using an inverted fluorescence microscope revealed that, compared to the original system (Figure 3), the dispersed phase exhibited a certain degree of coalescence (Figure 8).

**Figure 9:**
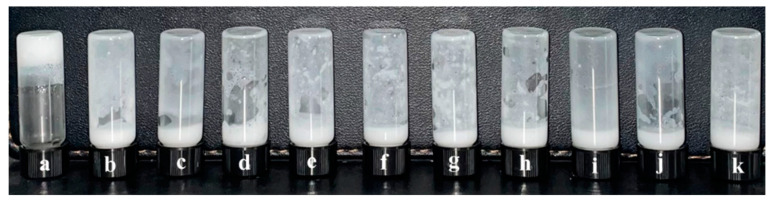
Photos of emulsions or gel emulsions formed at *N*-*t*-BAA concentrations ranging from 0% to 100% of the saturation concentration (relative to the organic phase): (**a**) 0%, (**b**) 10%, (**c**) 20%, (**d**) 30%, (**e**) 40%, (**f**) 50%, (**g**) 60%, (**h**) 70%, (**i**) 80%, (**j**) 90%, (**k**) 100% (stabilizer content is 2.5% (*w*/*v*) of the organic phase, D-PDMS is 0.075 g, and the aqueous phase makes up 90% of the total volume).

**Figure 10:**
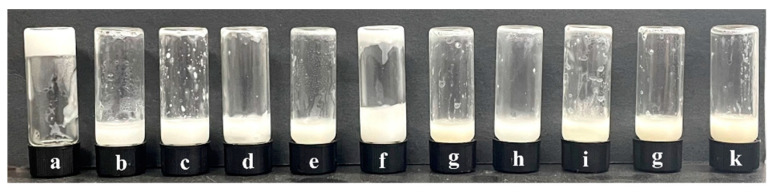
Photos of emulsions formed with different DCM–styrene (*v*/*v*) ratios: (**a**) 10:0, (**b**) 9:1, (**c**) 8:2, (**d**) 7:3, (**e**) 6:4, (**f**) 5:5, (**g**) 4:6, (**h**) 3:7, (**i**) 2:8, (**j**) 1:9, (**k**) 0:10 (stabilizer content is 2.5% (*w*/*v*) of the organic phase, D-PDMS is 0.075 g, and the aqueous phase makes up 90% of the total volume).

Similarly, inversion experiments (Figure 9) revealed that adding *N*-*t*-BAA failed to form a new gel emulsion system, completely disrupting the original gel emulsion system. Interestingly, a comparison of the molecular structures of the polymerizable monomers (Figure 4b,c) shows that *N*-*t*-BAA differs from *N*-*t*-BMA by only one methyl group. However, this structural difference enhanced the hydrophilicity of *N*-*t*-BAA, which completely destroyed the hydrophilic/lipophilic balance of the original gel emulsion system when introduced. A similar result was observed when styrene was added (Figure 10), as no new gel emulsion system could be formed. However, unlike *N*-*t*-BAA, styrene exhibited a dominant lipophilic nature. This characteristic also significantly disrupted the hydrophilic/lipophilic balance of the original system, rendering it incapable of maintaining its stable structure.

### 2.5. Rheological Properties

**Figure 11:**
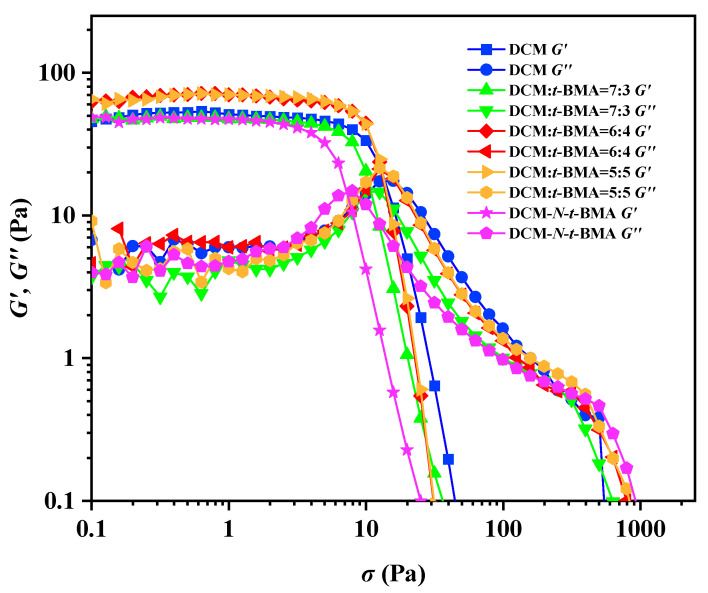
The *G*′ and *G*″ curves of DCM and gel emulsions containing different polymerizable monomers as a function of shear stress.

As a class of soft material, gel emulsions can have their stability quantitatively characterized by rheological tests (Figure 11). In this study, with the stabilizer content maintained at 2.5% (*w*/*v*) of the continuous phase, the storage modulus (*G*′) and loss modulus (*G*″) of the H_2_O/CSD-D-PDMS/DCM system and the gel emulsion systems incorporating different polymerizable monomers were measured as functions of shear stress. The rheological test results showed that, when the shear stress was below the yield stress, *G*′ was always greater than the corresponding *G*″, indicating solid-like behavior, i.e., elasticity. However, when the shear stress exceeded the yield stress, both *G*′ and *G*″ dropped sharply, and *G*″ became greater than *G*′, signifying fluid-like behavior, i.e., viscosity. This transition indicated the breakdown of the gel emulsion structure. As shown in Figure 11, for the base gel emulsion with DCM as the sole continuous phase, the *G*′, *G*″, and yield stress were 52 Pa, 6 Pa, and 13 Pa, respectively. For gel emulsions with DCM–*t*-BMA (*v*/*v*) ratios of 7:3, 6:4, and 5:5, their *G*′ and *G*″ values exhibited an increasing trend, measuring 45 Pa, 56 Pa, and 66 Pa, and 3 Pa, 8 Pa, and 9 Pa, respectively. The yield stress also increased at 10 Pa, 12 Pa, and 13 Pa, respectively. These results indicate that the content of *t*-BMA influences both the stability and viscoelasticity of the gel emulsion systems. Notably, gel emulsions with DCM–*t*-BMA (*v*/*v*) ratios of 6:4 or 5:5 had *G*′ and *G*″ values slightly higher than those of the base gel emulsion, whereas the system with a ratio of 7:3 exhibited slightly lower values. This demonstrates that adding an appropriate amount of *t*-BMA can enhance the stability of gel emulsions, while excessive or insufficient *t*-BMA reduces stability. In contrast, for gel emulsions with DCM-*N*-*t*-BMA as the continuous phase, the *G*′, *G*″, and yield stress were measured at 43 Pa, 3 Pa, and 7 Pa, respectively, all of which were lower than or equal to those of other systems, indicating that this system had the weakest stability. The trends in gel emulsion stability revealed by rheological analysis are consistent with the inversion test results (Figure 5, Figure 7, Figure 9 and Figure 10). Comparing the molecular structures of *t*-BMA and *N*-*t*-BMA (Figure 4a,b) with the experimental results, it becomes evident that even minor structural modifications to polymerizable monomers can significantly affect the stability of gel emulsion systems.

## 3. Experimental Section

### 3.1. Reagents and Instruments

The 1,3,5-tribromobenzene (≥98%), 4-ethynylaniline (≥97%), tetrakis(triphenylphosphine)palladium (≥99%), cholesterol chloroformate (≥98%), diisopropylamine (≥98%) were purchased from Shanghai Merial Biotech Co., Ltd. (Merial Biotech, Shanghai, China); copper(I) iodide (≥98%) was purchased from Beijing Bailingwei Technology Co., Ltd. (Beijing Bailingwei Technology, Beijing, China); triethylamine (≥99.5%) was obtained from SAEN Chemical Technology (Shanghai, China) Co., Ltd. (SAEN Chemical Technology, Shanghai, China); NH_2_-PDMS-NH_2_ (M_n_ = 900–1000) was purchased from Gelest Inc. (Gelest, Morrisville, PA, USA); acryloyl chloride (≥96%) was purchased from Shanghai Aladdin Biochemical Technology Co., Ltd. (Aladdin Biochemical Technology, Shanghai, China). All chemicals were used without further purification. Tetrahydrofuran was distilled after refluxing with sodium pieces; *n*-hexane, methanol, ethyl acetate, and other organic solvents were distilled and purified before use; laboratory water was ultrapure water purified by the UP ultrapure water system.

SCR1-type thermostatic magnetic stirrer (BIOCOTE Ltd., Coventry, UK); C20-type electric thermostatic drying oven (Shanghai Ledon Industrial Co., Ltd., Shanghai, China); ADVANCE 600 fourier digital NMR spectrometer (BRUKER, Fällanden, Switzerland); VERTEX70 infrared spectrometer (BRUKER, Fällanden, Switzerland); high-resolution mass spectra (Thermo Fisher Scientific, Waltham, MA, USA); ARES-G2 rheometer (TA Instruments, New Castle, DE, USA); Vortex3000 vortex shaker (WIGGENS, Wuppertal, Germany); Olympus IX73 inverted fluorescence microscope (OLYMPUS, Tokyo, Japan).

### 3.2. Experimental Procedure

#### 3.2.1. Synthesis and Characterization of CSD and D-PDMS

**Scheme 1:**
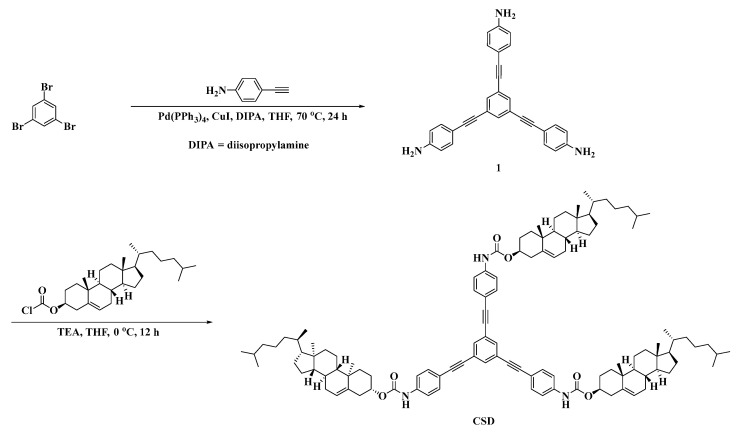
Synthesis route of the CSD.

The synthesis process of the stabilizer CSD is shown in Scheme 1.

##### Synthesis of Intermediate 1

Weigh 0.25 g (0.79 mmol) of 1,3,5-tribromobenzene, 0.36 g (3 mmol) of 4-ethynylaniline, 91 mg (0.079 mmol) of tetrakis(triphenylphosphine)palladium, and 15 mg (0.079 mmol) of copper(I) iodide in a 120 mL dry pressure bottle. Add 20 mL of anhydrous diisopropylamine and 30 mL of tetrahydrofuran, seal the pressure bottle, heat to 70 °C, and stir the reaction for 24 h, monitoring the reaction using TLC. After the reaction is complete, cool to room temperature, dilute the reaction mixture with 100 mL of DCM, and wash successively with saturated ammonium chloride solution, sodium bicarbonate solution, and saturated sodium chloride solution. After drying with anhydrous Na_2_SO_4_, remove the organic phase by rotary evaporation. The crude product is purified by column chromatography (petroleum ether/ethyl acetate, 1/1) to yield a light yellow solid, which is intermediate 1, with a yield of 65% [35].

##### Synthesis of CSD

At 0 °C, dissolve 1.70 g (3.80 mmol) of cholesterol chloroformate in a flask containing 30 mL of tetrahydrofuran. Then, dissolve 0.43 mg (1 mmol) of intermediate 1 and 530 µL (3.80 mmol) of triethylamine in 20 mL of tetrahydrofuran and add this solution dropwise to the cholesterol chloroformate solution using a pressure-equalizing dropping funnel. Stir the resulting mixture under an ice bath for 1 h, then allow it to return to room temperature and continue stirring for 12 h. After the reaction is complete, dilute the reaction mixture with DCM, wash with saturated sodium chloride solution, dry with anhydrous Na_2_SO_4_, and remove the organic phase by rotary evaporation. The crude product is purified by column chromatography (petroleum ether/DCM, 1/1) to obtain a light yellow solid, which is CSD, with a yield of 78%.

##### Synthesis of Crosslinker D-PDMS

**Scheme 2:**
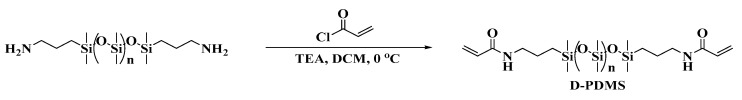
Synthesis route of the D-PDMS.

The synthesis process of the crosslinking agent is shown in Scheme 2.

At 0 °C, measure 0.25 mL (3.0 mmol) of acryloyl chloride and dissolve it in a flask containing 20 mL of DCM. Dissolve 1 g (approximately 1.0 mmol) of NH_2_-PDMS-NH_2_ (M_n_ = 900–1000) and 0.15 mL (1.0 mmol) of triethylamine in 90 mL of DCM and add this solution dropwise to the acryloyl chloride solution using a pressure-equalizing dropping funnel. Stir the resulting mixture under an ice bath for 12 h. After the reaction is complete, wash the reaction mixture with saturated saline solution five times, dry with anhydrous Na_2_SO_4_, and remove the solvent by rotary evaporation to obtain a transparent oily liquid, which is the target product D-PDMS, with a yield of 95%.

##### Structural Characterization of the Compounds

Intermediate 1. ^1^H NMR (600 MHz, DMSO/TMS) δ 7.44 (s, 1H, Ar), 7.29–7.17 (d, 2H, Ar), 6.66–6.48 (d, 2H, Ar), 5.64 (s, 2H, NH_2_). ^13^C NMR (150 MHz, DMSO/TMS) δ 150.32, 133.30, 132.04, 125.04, 114.09, 108.03, 93.21, 85.65. IR: 3350 cm^−1^ (N-H), 2190 cm^−1^ (C☰C). HRMS (ESI-TOF): *m*/*z* [M + H]^+^ calcd. for C_30_H_22_N_3_^+^ 424.18137, found 424.17981. M.p.: 138.6 °C.

Stabilizer (CSD). ^1^H NMR (600 MHz, CDCl_3_/TMS) δ 7.57 (s, 1H, Ar), 7.46–7.42 (m, 2H, Ar), 7.37 (d, 2H, Ar), 6.67 (s, 1H, NHCO), 5.39 (dt, 1H, alkenyl), 4.60 (tt, 1H, oxycyclohexyl), 0.66–2.40 (m, 43H, cholesteryl protons). ^13^C NMR (150 MHz, CDCl_3_/TMS) δ 152.87, 139.56, 138.48, 133.75, 132.71, 124.18, 123.00, 118.30, 117.44, 90.42, 87.46, 75.31, 56.75, 56.22, 50.04, 42.39, 39.80, 39.61, 38.52, 37.03, 36.65, 36.27, 35.92, 32.00, 31.93, 28.34, 28.16, 28.12, 24.39, 23.97, 22.95, 22.69, 21.13, 19.44, 18.81, 11.96. IR: 3340 cm^−1^ (N-H), 2190 cm^−1^ (C☰C), 1730 cm^−1^ (C=O), 1620 cm^−1^ (C=C). HRMS (ESI-TOF): *m*/*z* [M + Na]^+^ calcd. for C_114_H_153_N_3_O_6_Na^+^ 1684.16906, found 1684.03215. M.p.: 264.8 °C.

Crosslinker (D-PDMS). ^1^H NMR (600 MHz, CDCl_3_/TMS) δ 6.20 (dd, 1H, alkenyl), 6.06–5.97 (m, 1H, alkenyl), 5.55 (dd, 1H, alkenyl), 3.24 (p, 2H, CH_2_NH), 1.55–1.47 (m, 2H, CH_2_CH_2_), 0.53–0.42 (m, 2H, CH_2_Si(CH_3_)). ^13^C NMR (150 MHz, CDCl_3_/TMS) δ 164.39, 129.98, 125.11, 41.39, 22.47, 14.39, 0.24, 0.14. IR: 3280 cm^−1^ (N-H), 1660 cm^−1^ (C=O), 1620 cm^−1^ (C=C).

#### 3.2.2. Gelation Experiment

Weigh 0.0175 g of the stabilizer and 0.5 mL of the test gel solvent in a sealed sample bottle (D = 1 cm, H = 3 cm). After ultrasonic treatment at room temperature (20 °C) for 20 min (ultrasound frequency 40 kHz, power 150 W), let it stand and invert the sample bottle to check if a gel is formed, or heat it until the solid completely dissolves, then cool it to room temperature and invert the sample bottle again to observe if gelation occurs. If the gel forms after ultrasound, record it as G (gel). If the substance forms a solution upon ultrasound or heating, record it as S (solution). For systems that dissolve upon heating but precipitate upon cooling, record it as P (precipitate). A mixture of gel and solution is recorded as PG (partial gel). If the system does not dissolve even when heated to the boiling point of the solvent, record it as I (insoluble) [22]. The specific time of ultrasound-induced gelation is shown in Table 2.

#### 3.2.3. Preparation of Gel Emulsion

Add CSD (at a concentration of 2.5% continuous phase, *w*/*v*), 0.075 g of D-PDMS, and the polymerizable monomers into a sample bottle (D = 1 cm, H = 3 cm). Then, add a certain amount of the oil phase (100 µL, 10% of the total volume of the solvent system, *v*/*v*) to the sample bottle. After CSD and D-PDMS have completely dissolved at room temperature or mild heat, gradually add the corresponding amount of ultrapure water (900 µL, 90% of the total volume of the solvent system, *v*/*v*) in the appropriate ratio while shaking until the process is complete. Finally, subject the mixture to intense shaking at a constant speed of 2500 rpm using a homogenizer to thoroughly mix the oil and water phases and form a viscous solution. After letting it stand for about 3 min, invert the test tube, and, if it no longer flows, the gel emulsion is obtained. The compositions of the gel emulsions are listed in Table 3.

#### 3.2.4. *T*_gel_ Measurements

After preparing the gel in a small pressure-resistant tube, a small steel ball weighing less than 100 mg is placed on top of the gel using magnetic manipulation. The gel is then heated in an oil bath at a heating rate of 1 °C/min. When the gel transitions into a sol, the steel ball begins to move downward, and the gel’s phase transition temperature (*T*_gel_) is determined simultaneously [36].

#### 3.2.5. Rheological Measurements

The rheological properties of the gel emulsions were measured using a stress-controlled rheometer (TA Instruments, ARES-G2) equipped with a geometrically constructed steel-coated parallel plate (20 mm in diameter). The gap between the two plates was set to 1.0 mm, and the temperature was maintained at 20 °C. A solvent-trapping device was placed above the plate to minimize solvent evaporation during the measurement. A stress sweep at a constant frequency (1 Hz) was performed, which provided information about the linear viscoelastic region of the gel emulsions sample.

#### 3.2.6. Inverted Fluorescence Microscope Observation

**Figure 12:**
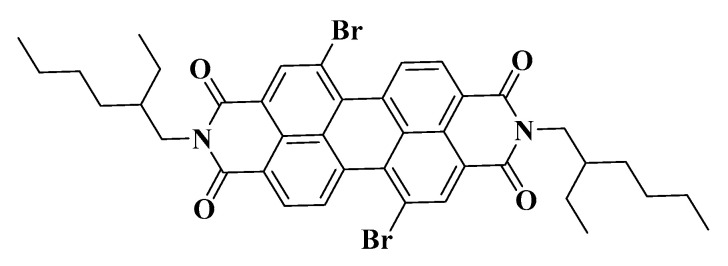
Molecular structure of the fluorescent probe.

An organic solution containing 1 × 10^−4^ M fluorescent probe was used as the continuous phase to prepare the gel emulsion, which was allowed to stand for 12 h before testing. The microstructure of the gel emulsion was observed using an Olympus IX73 inverted fluorescence microscope from Olympus, Japan. The fluorescent probe used was 5,12-dibromo-2,9-bis(2-ethylhexyl)anthracene[2,1,9-DEF:6,5,10-d’e’f’]diquinoline-1,3,8,10(2*H*,9*H*)-tetraone (Figure 12), with excitation and emission wavelengths of 365 nm and 550 nm, respectively.

## 4. Conclusions

This research, based on a W/O gel emulsion synergistically stabilized by an LMMG and a cross-linker, successfully developed two novel in situ polymerizable gel emulsion systems by introducing suitable amphiphilic polymerizable monomers. These two novel gel emulsion systems lay the foundation for further achieving the preparation of functionalized POPs using them as templates. This research found that the balance of amphiphilicity of the polymerizable monomers played a decisive role in maintaining the stability of the new gel emulsion system, with small structural changes in the monomers leading to significantly different experimental results. This research demonstrates that developing novel gel emulsion systems through the synergistic effect of LMMGs and cross-linkers in a solvent system is reasonable and feasible. The findings provide an easily implementable and effective model for developing new polymerizable gel emulsion systems and templated preparation of POPs. In future studies, we will use the obtained gel emulsions as a template to prepare POPs and evaluate their performance in adsorption and separation. Additionally, we will explore their applications in luminescent materials and other fields.

## Figures and Tables

**Table 1:** CSD behavior of gelator (3.5%, *w*/*v*) in different solvents; gel–sol phase transition temperature (*T*_gel_/°C).

Solvent	Result	Solvent	Result		Solvent	Result	
methanol	P	petroleum ether	G, ~74 ^a^	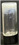	*n*-hexane	G, ~45	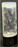
ethanol	P	DCM	G, ~50	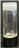	*n*-heptane	G, ~41	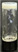
*n*-propanol	P	ethyl acetate	G, ~84	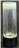	*n*-octane	G, ~36	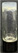
*n*-butanol	P	CCl_4_	G, ~42	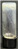	*n*-nonane	G, ~54	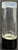
*n*-pentanol	P	TEA	G, ~94	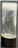	*n*-decane	G, ~49	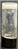
isopropanol	P	*t*-BMA	G, ~112	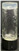	cyclohexane	G, ~98	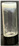
vinyl cyanide	P	MMA	PG		toluene	G, ~46	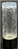
pyridine	S	ethyl ether	PG		styrene	G, ~78	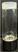
CHCl_3_	S	DMSO	P		isopentanol	P	
H_2_O	I	DMF	P		acetone	P	
acetic acid	I	THF	S		acetonitrile	I	

S = Solution; P = Precipitation; I = Insoluble; G = Gel; PG = Partial Gel. DCM is short for dichloromethane; *t*-BMA is short for *tert*-butyl methacrylate; MMA is short for methyl methacrylate. ^a^ 74 means that the *T*_gel_ (gel–sol phase transition temperature) of the gel system is 74 °C.

**Table 2:** Gelation time of CSD in different organic solvents under ultrasound-induced (40 kHz, 150 W).

Solvent	Time (Result)	Solvent	Time (Result)	Solvent	Time (Result)	Solvent	Time (Result)
petroleum ether	3 min (G)	TEA	5 min (G)	*n*-hexane	10 min (G)	*n*-decane	15 min (G)
DCM	2 min (G)	*t*-BMA	4 min (G)	*n*-heptane	10 min (G)	cyclohexane	8 min (G)
ethyl acetate	5 min (G)	MMA	20 min (PG)	*n*-octane	15 min (G)	toluene	6 min (G)
CCl_4_	3 min (G)	ethyl ether	20 min (PG)	*n*-nonane	15 min (G)	styrene	4 min (G)

G = Gel; PG = Partial Gel.

**Table 3:** Compositions of the gel emulsions.

NO.	CSD (%, *w*/*v*)	DCM (µL)	D-PDMS (g)	Water (µL)	*t*-BMA (µL)	*N*-*t*-BMA (g)	Result
CSD_2.5-0_ ^a^	2.5	100	0.075	900	0	0	GE
CSD_2.5-30 (l)_	2.5	70	0.075	900	30	0	GE
CSD_2.5-40 (l)_	2.5	60	0.075	900	40	0	GE
CSD_2.5-50 (l)_	2.5	50	0.075	900	50	0	GE
CSD_2.5-50 (s)_^b^	2.5	100	0.075	900	0	0.05	GE

^a^ CSD *_x-y_*_(monomer state)_, where *x* stands for the concentration of CSD in the organic phase and *y* the volume fraction of the polymerizable monomer (l) or percentage relative to the saturation concentration of the polymerizable monomer (s) in 100 μL of DCM; ^b^ the saturation solubility of *N*-*t*-BMA in 100 µL of DCM is 0.1 g. GE stands for gel emulsion.

## Data Availability

Data are contained within the article and Supplementary Materials.

## Supplementary Materials

The following supporting information can be downloaded at https://www.mdpi.com/article/10.3390/molecules30040787/s1, Figure S1: ^1^H NMR spectrum of intermediate 1; Figure S2: ^13^C NMR spectrum of intermediate 1; Figure S3: ^1^H NMR spectrum of CSD; Figure S4: ^13^C NMR spectrum of CSD; Figure S5: ^1^H NMR spectrum of D-PDMS; Figure S6: ^13^C NMR spectrum of D-PDMS; Figure S7: The FTIR spectra of intermediate 1 and CSD; Figure S8: The FTIR spectra of D-PDMS; Figure S9: ESI mass spectrum of intermediate 1; Figure S10: ESI mass spectrum of CSD. Reference [35] is cited in the Supplementary Materials.
