# Supplementary material for: The Development and Preparation of Novel Gel Emulsion Systems Based on a Cholesterol Star-Shaped Derivative
Source: Molecules. 2025 Feb 8;30(4):787. doi: 10.3390/molecules30040787 (PMC11857901; doi:10.3390/molecules30040787)
Supplement: Supplementary file 1 [file molecules-30-00787-s001.zip › molecules-3444790-supplementary.pdf]

Supplementary Material

for

The Development and Preparation of Novel Gel Emulsion Systems Based on a Cholesterol Star-Shaped Derivative

Shuaihua Liu [1], Tian Yao [1], Donghui Xia [1,2], Quan Liu [1,2], Guanghui Tian [1,2] and Yang Liu [1,2,*]

[1] School of Chemistry and Environment Science, Shaanxi University of Technology, Hanzhong 723001, China

[2] Shaanxi Key Laboratory of Catalysis, Shaanxi University of Technology, Hanzhong 723001, China

* Correspondence: liuyang@snut.edu.cn (Y.L.)

CONTENS

1. Experimental Section...……………………………………………………1

2. Supplementary Figures…………………………………………………2

3. References……………………………………………………………7

1. Experimental Section

1.1. Reagents and materials

1,3,5-tribromobenzene (≥98%), 4-ethynylaniline (≥97%), tetrakis(triphenylphosphine)palladium (≥99%), cholesterol chloroformate (≥98%), diisopropylamine (≥98%) were purchased from Shanghai Merial Biotech Co., Ltd. (Merial Biotech, Shanghai, China); copper(I) iodide (≥98%) was purchased from Beijing Bailingwei Technology Co., Ltd. (Beijing Bailingwei Technology, Beijing, China); triethylamine (≥99.5%) was obtained from SAEN Chemical Technology (Shanghai) Co., Ltd. (SAEN Chemical Technology, Shanghai, China); NH_2-PDMS-NH_2 (M_n = 900-1000) was purchased from Gelest Inc.(Gelest, Morrisville, PA, USA); acryloyl chloride (≥96%) was purchased from Shanghai Aladdin Biochemical Technology Co., Ltd. (Aladdin Biochemical Technology, Shanghai, China). All chemicals were used without further purification. Tetrahydrofuran was distilled after refluxing with sodium pieces; *n*-hexane, methanol, ethyl acetate, and other organic solvents were distilled and purified before use; laboratory water was ultrapure water purified by the UP ultrapure water system.

1.2. Preparation of intermediate 1 and CSD

Synthesis of intermediate 1: Weigh 0.25 g (0.79 mmol) of 1,3,5-tribromobenzene, 0.36 g (3 mmol) of 4-ethynylaniline, 91 mg (0.079 mmol) of tetrakis(triphenylphosphine)palladium, and 15 mg (0.079 mmol) of copper(I) iodide into a 120 mL dry pressure bottle. Add 20 mL of anhydrous diisopropylamine and 30 mL of tetrahydrofuran, seal the pressure bottle, heat to 70 °C, and stir the reaction for 24 h, monitoring the reaction using TLC. After the reaction is complete, cool to room temperature, dilute the reaction mixture with 100 mL of dichloromethane, and wash successively with saturated ammonium chloride solution, sodium bicarbonate solution, and saturated sodium chloride solution. After drying with anhydrous sodium sulfate, remove the organic phase by rotary evaporation. The crude product is purified by column chromatography (petroleum ether/ethyl acetate, 1/1) to yield a light yellow solid, which is the intermediate 1, with a yield of 65%. ^{1}H NMR (600 MHz, DMSO/TMS) δ 7.44 (s, 1H, Ar), 7.29-7.17 (d, 2H, Ar), 6.66-6.48 (d, 2H, Ar), 5.64 (s, 2H, NH_2). ^{13}C NMR (150 MHz, DMSO/TMS) δ 150.32, 133.30, 132.04, 125.04, 114.09, 108.03, 93.21, 85.65. IR: 3350 cm^{-1} (N-H), 2190 cm^{-1} (C≡C). HRMS (ESI-TOF): *m/z* $[M + H]^+$ calcd. for $C_{30}H_{22}N_3^+$ 424.18137, found 424.17981. M.p.: 138.6 °C.

Synthesis of CSD: At 0 °C, dissolve 1.70 g (3.80 mmol) of cholesterol chloroformate in a flask containing 30 mL of tetrahydrofuran. Then, dissolve 0.43 mg (1 mmol) of intermediate 1 and 530 μL (3.80 mmol) of triethylamine in 20 mL of tetrahydrofuran and add this solution dropwise to the cholesterol chloroformate solution using a pressure-equalizing dropping funnel. Stir the resulting mixture under an ice bath for 1 h, then allow it to return to room temperature and continue stirring for 12 h. After the reaction is complete, dilute the reaction mixture with dichloromethane, wash with saturated sodium chloride solution, dry with anhydrous

Na_2SO_4, and remove the organic phase by rotary evaporation. The crude product is purified by column chromatography (petroleum ether/dichloromethane, 1/1) to obtain a light yellow solid, which is CSD, with a yield of 78%. ^1H NMR (600 MHz, $CDCl_3$/TMS) δ 7.57 (s, 1H, Ar), 7.46-7.42 (m, 2H, Ar), 7.37 (d, 2H, Ar), 6.67 (s, 1H, NHCO), 5.39 (dt, 1H, alkenyl), 4.60 (tt, 1H, oxycyclohexyl), 0.66-2.40 (m, 43H, cholesteryl protons). ^{13}C NMR (150 MHz, $CDCl_3$/TMS) δ 152.87, 139.56, 138.48, 133.75, 132.71, 124.18, 123.00, 118.30, 117.44, 90.42, 87.46, 75.31, 56.75, 56.22, 50.04, 42.39, 39.80, 39.61, 38.52, 37.03, 36.65, 36.27, 35.92, 32.00, 31.93, 28.34, 28.16, 28.12, 24.39, 23.97, 22.95, 22.69, 21.13, 19.44, 18.81, 11.96. IR: 3340 cm^{-1} (N-H), 2190 cm^{-1} (C≡C), 1730 cm^{-1} (C=O), 1620 cm^{-1} (C=C). HRMS (ESI-TOF): *m/z* $[M + Na]^+$ calcd. for $C_{114}H_{153}N_3O_6Na^+$ 1684.16906, found 1684.03215. M.p.: 264.8 °C.

1.3. Preparation of D-PDMS

At 0 °C, measure 0.25 mL (3.0 mmol) of acryloyl chloride and dissolve it in a flask containing 20 mL of dichloromethane. Dissolve 1 g (approximately 1.0 mmol) of NH_2-PDMS-NH_2 (M_n = 900-1000) and 0.15 mL (1.0 mmol) of triethylamine in 90 mL of dichloromethane and add this solution dropwise to the acryloyl chloride solution using a pressure-equalizing dropping funnel. Stir the resulting mixture under an ice bath for 12 h. After the reaction is complete, wash the reaction mixture with saturated saline solution five times, dry with anhydrous Na_2SO_4, and remove the solvent by rotary evaporation to obtain a transparent oily liquid, which is the target product D-PDMS, with a yield of 95%. ^1H NMR (600 MHz, $CDCl_3$/TMS) δ 6.20 (dd, 1H, alkenyl), 6.06-5.97 (m, 1H, alkenyl), 5.55 (dd, 1H, alkenyl), 3.24 (p, 2H, CH_2NH), 1.55-1.47 (m, 2H, CH_2CH_2), 0.53-0.42 (m, 2H, $CH_2Si(CH_3)$). ^{13}C NMR (150 MHz, $CDCl_3$/TMS) δ 164.39, 129.98, 125.11, 41.39, 22.47, 14.39, 0.24, 0.14. IR: 3280 cm^{-1} (N-H), 1660 cm^{-1} (C=O), 1620 cm^{-1} (C=C).

2. Supplementary Figures

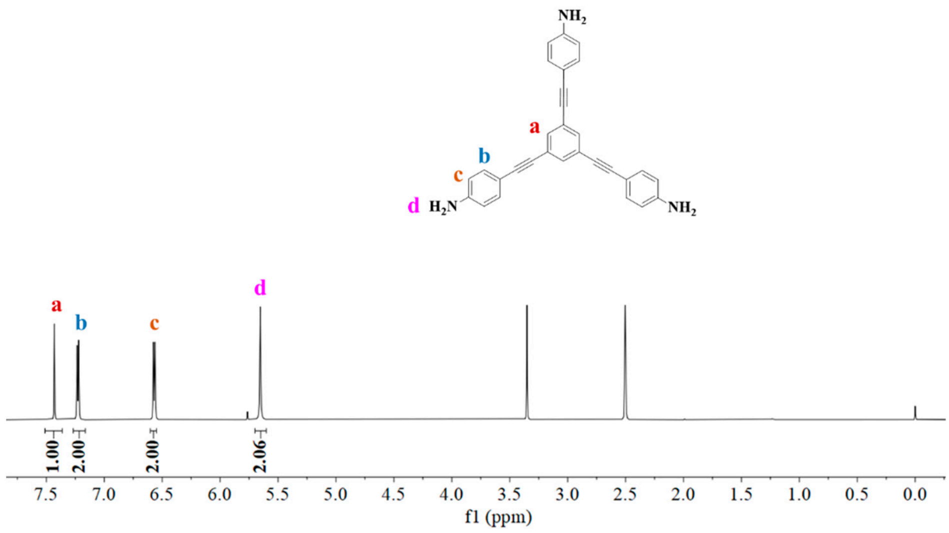

Figure S1. ^1H NMR spectrum of intermediate 1.

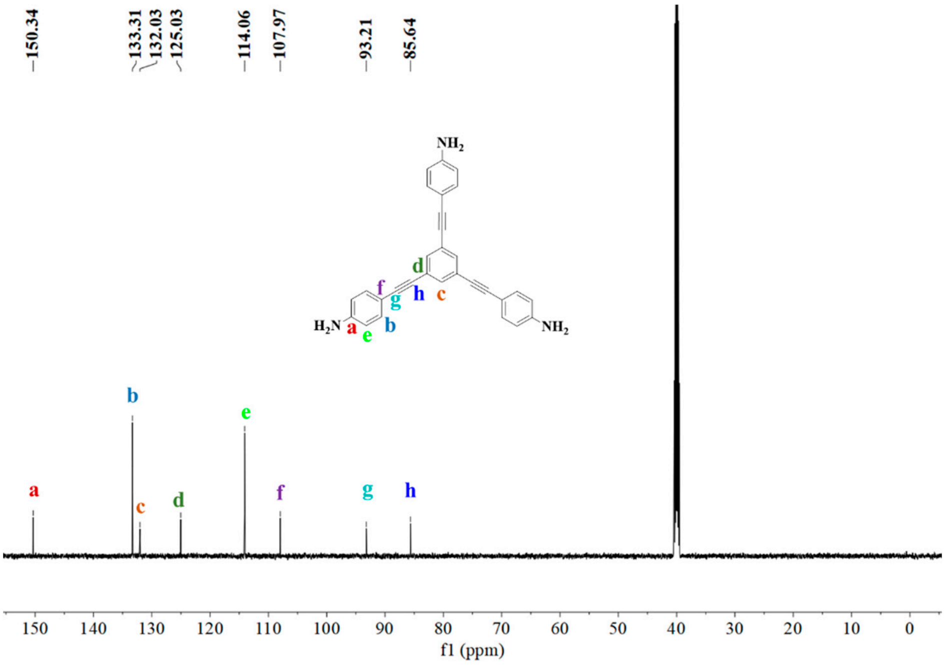

Figure S2. ^{13}C NMR spectrum of intermediate 1.

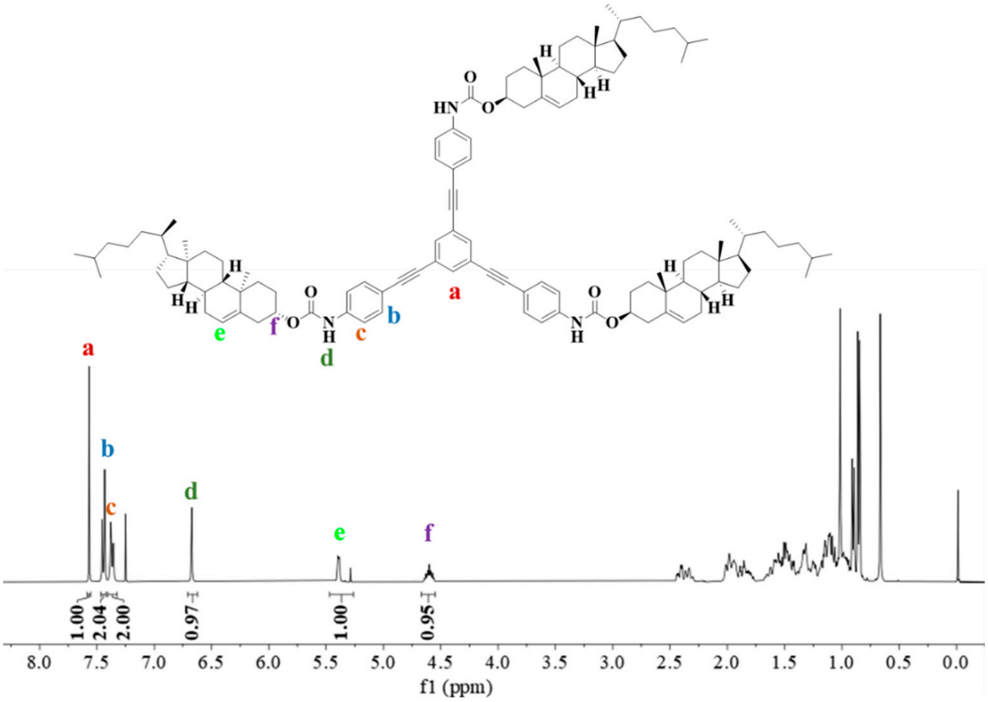

Figure S3. ^{1}H NMR spectrum of CSD.

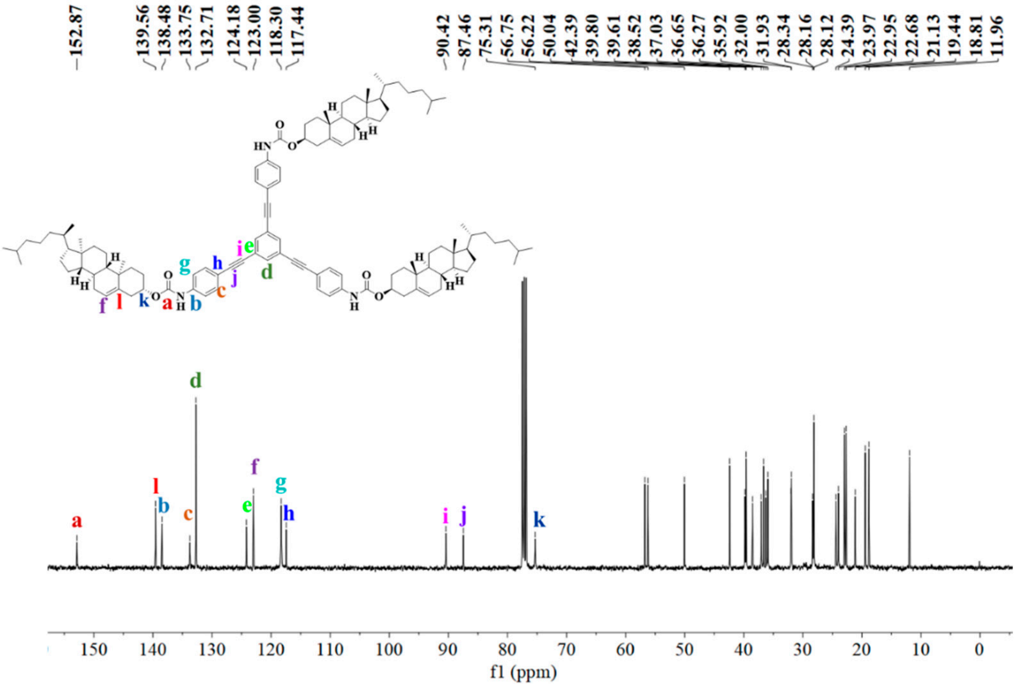

Figure S4. ^{13}C NMR spectrum of CSD.

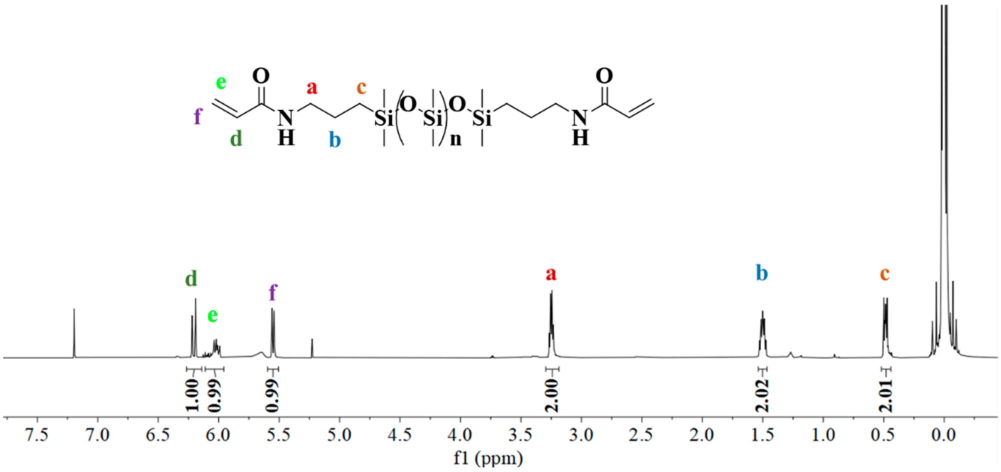

Figure S5. ^{1}H NMR spectrum of D-PDMS.

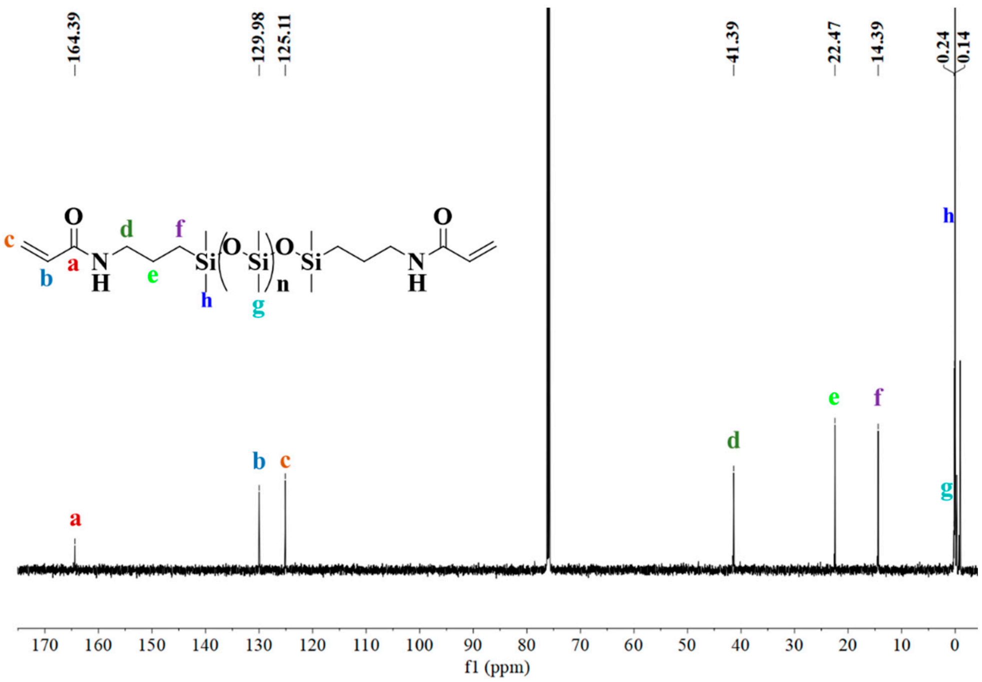

Figure S6. ^{13}C NMR spectrum of D-PDMS.

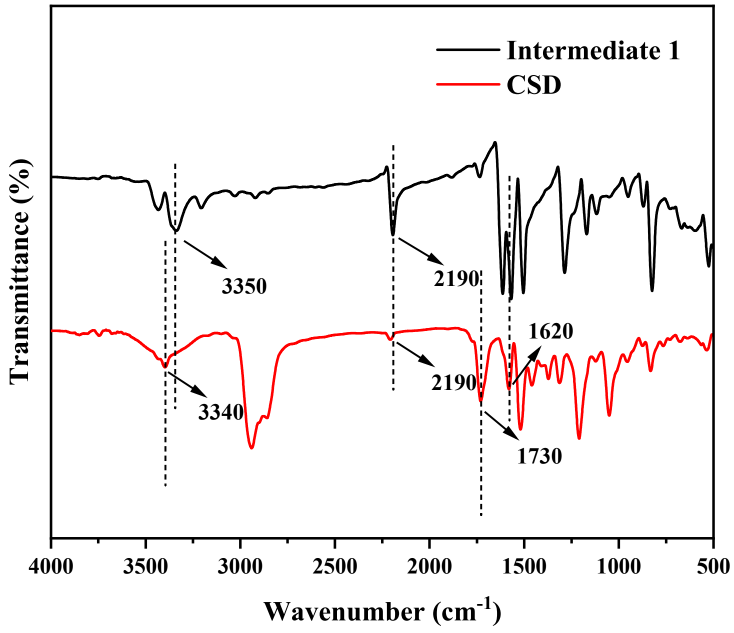

Figure S7. The FTIR spectra of intermediate 1 and CSD.

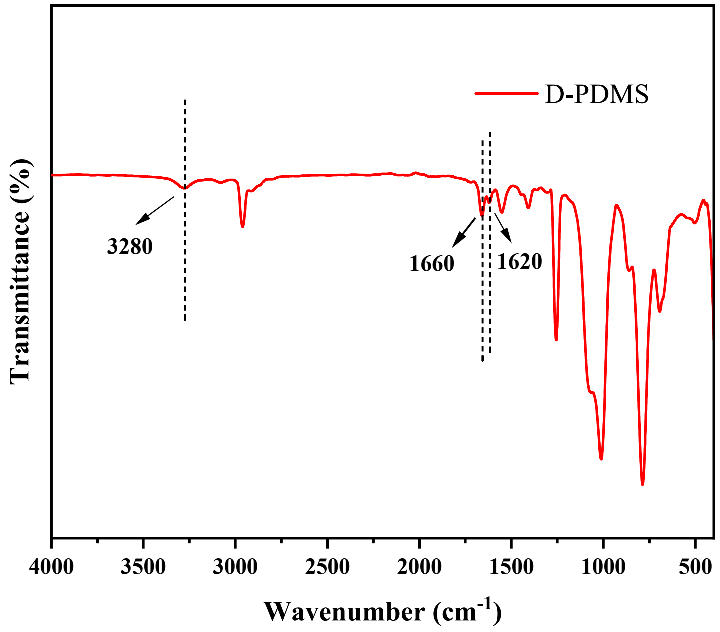

Figure S8. The FTIR spectra of D-PDMS.

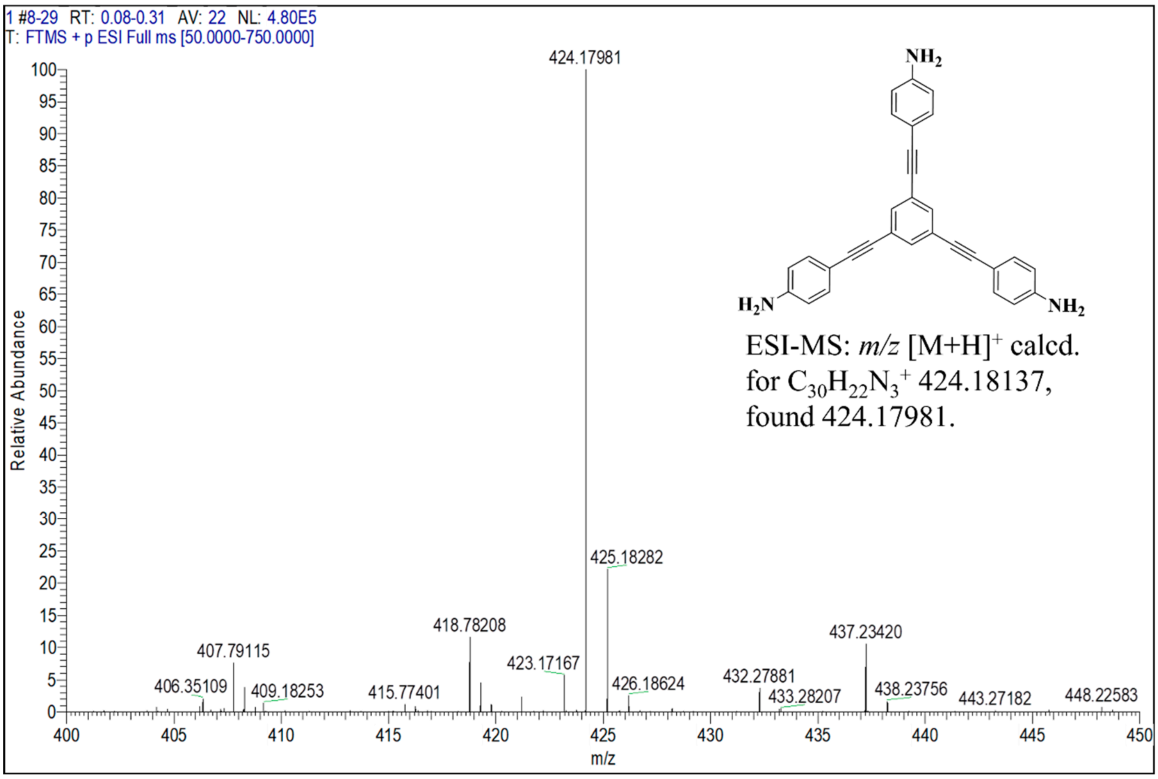

Figure S9. ESI mass spectrum of intermediate 1.

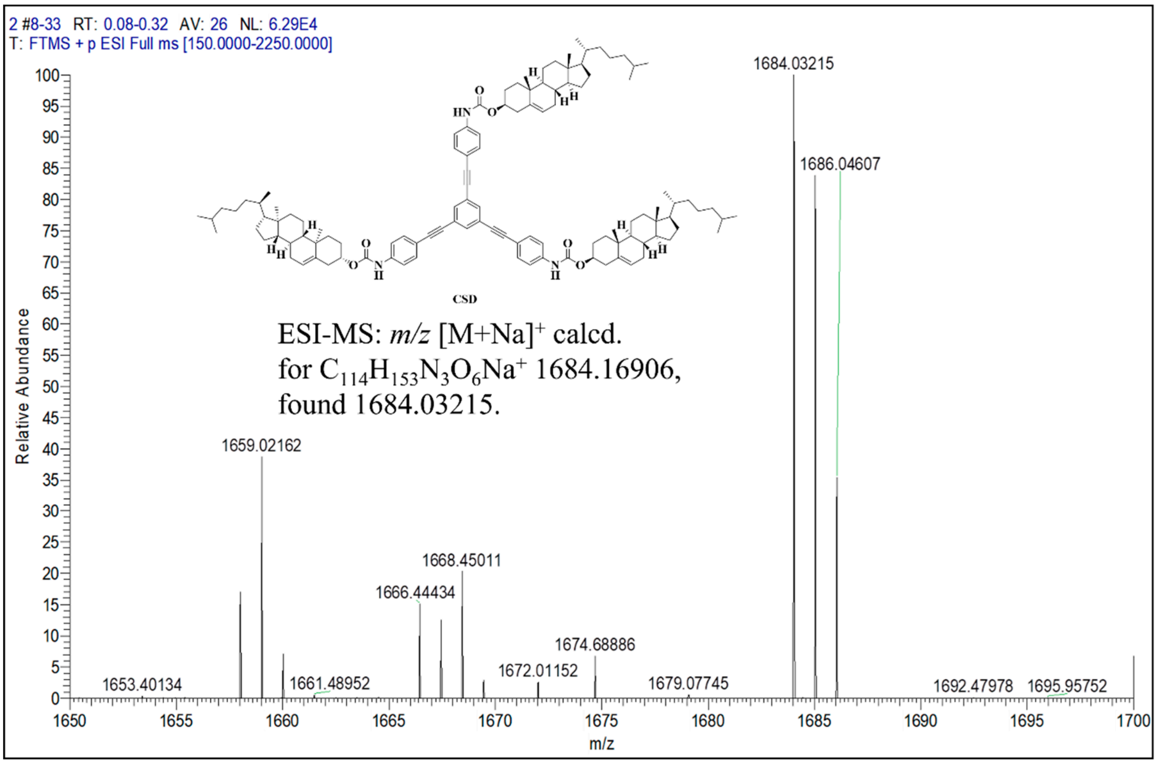

Figure S10. ESI mass spectrum of CSD.

3. References

1 Jagadesan, P.; Whittemore, T.; Beirl, T.; Turro, C.; McGrier, P. L. Excited-state intramolecular proton-transfer properties of three tris(*N*-salicylideneaniline)-based chromophores with extended conjugation. *Chem. Eur. J.* **2017**, *23*, 917-925.
